# Functional Traits Reveal Processes Driving Natural Afforestation at Large Spatial Scales

**DOI:** 10.1371/journal.pone.0075219

**Published:** 2013-09-18

**Authors:** Norman W. H. Mason, Susan K. Wiser, Sarah J. Richardson, Michael J. Thorsen, Robert J. Holdaway, Stéphane Dray, Fiona J. Thomson, Fiona E. Carswell

**Affiliations:** 1 Landcare Research, Hamilton, New Zealand; 2 Landcare Research, Lincoln, New Zealand; 3 Department of Botany, University of Otago, Dunedin, New Zealand; 4 Université Lyon 1, CNRS, UMR5558, Laboratoire de Biométrie et Biologie Evolutive, F-69622, Villeurbanne, France; Norwegian University of Science and Technology, Norway

## Abstract

An understanding of the processes governing natural afforestation over large spatial scales is vital for enhancing forest carbon sequestration. Models of tree species occurrence probability in non-forest vegetation could potentially identify the primary variables determining natural afforestation. However, inferring processes governing afforestation using tree species occurrence is potentially problematic, since it is impossible to know whether observed occurrences are due to recruitment or persistence of existing trees following disturbance. Plant functional traits have the potential to reveal the processes by which key environmental and land cover variables influence afforestation. We used 10,061 survey plots to identify the primary environmental and land cover variables influencing tree occurrence probability in non-forest vegetation in New Zealand. We also examined how these variables influenced diversity of functional traits linked to plant ecological strategy and dispersal ability. Mean annual temperature was the most important environmental predictor of tree occurrence. Local woody cover and distance to forest were the most important land cover variables. Relationships between these variables and ecological strategy traits revealed a trade-off between ability to compete for light and colonize sites that were marginal for tree occurrence. Biotically dispersed species occurred less frequently with declining temperature and local woody cover, suggesting that abiotic stress limited their establishment and that biotic dispersal did not increase ability to colonize non-woody vegetation. Functional diversity for ecological strategy traits declined with declining temperature and woody cover and increasing distance to forest. Functional diversity for dispersal traits showed the opposite trend. This suggests that low temperatures and woody cover and high distance to forest may limit tree species establishment through filtering on ecological strategy traits, but not on dispersal traits. This study shows that ‘snapshot’ survey plot data, combined with functional trait data, may reveal the processes driving tree species establishment in non-forest vegetation over large spatial scales.

## Introduction

Succession of non-forest vegetation to forest has made a significant contribution to global carbon sequestration in recent decades [1,2]. An understanding of the factors and processes governing natural afforestation over large spatial scales (i.e. hundreds of thousands of square kilometers) is vital for enhancing forest carbon sequestration. Models of tree occurrence probability in non-forest vegetation could potentially identify the primary environmental and land cover variables determining natural afforestation. However, inferring processes governing afforestation using tree species occurrence is potentially problematic, since it is impossible to know whether observed occurrences are due to recruitment or to persistence of existing trees following disturbance. Plant functional traits can reveal the processes driving forest succession following disturbance [3,4]. They have the potential to complement the patterns revealed by predictive modeling, by helping us to understand the processes by which key environmental and land cover variables in predictive models influence tree species occurrence. However, it remains unclear whether this is true over very large scales. This study models tree species occurrence probability in national-scale ‘snapshot’ non-forest survey plots to reveal the primary land cover and environmental variables influencing tree species occurrence. It then explores the potential of traits linked to plant ecological strategy (i.e. adaptations influencing species’ ability to establish and compete at a given site) and seed dispersal ability to reveal the processes by which these site variables influence tree species occurrence.

Three of the main factors influencing tree species occurrence in non-forest vegetation are abiotic stress (especially temperature extremes and water limitation), local vegetation cover (particularly the degree of woodiness) and proximity to seed source. Abiotic stress limits establishment by increasing tree seedling mortality and excluding stress-intolerant species [5-7]. Woody cover may promote establishment by alleviating abiotic stress (especially drought and frost, e.g. [5,8,9]), protecting palatable tree species from ungulate herbivory (e.g. [10-12]) and enhancing biotic seed dispersal (especially by birds [13-15]), or through a combination of these effects [16]. Distance from seed source may affect not only the number of seeds arriving but also the range of species able to colonize a site, since only species with high dispersal ability will be able to colonize sites distant from seed source [17].

### Functional traits and processes driving tree species occurrences

Functional traits relating to ecological strategy are increasingly recognized as a useful means for understanding the processes driving changes in species occurrences and abundances along landscape-scale gradients of environmental stress (e.g. [18-20]) and succession (e.g. [21,22]). However, studies of changes in dispersal traits along gradients are much less common (but see [23]), even though the potential for differences in regeneration strategy to promote species co-existence in plant communities has long been recognized [24].

Ecological strategy traits are those that relate to a species’ successional status and resource use strategy (e.g. [25,26]). Leaf traits are emerging as highly effective indicators of plant resource use strategy [27], providing a means of contrasting ‘slow and tight’ stress tolerators (i.e. high leaf longevity and low photosynthetic rates) against ‘fast and leaky’ resource acquirers (rapid leaf turnover and high photosynthetic rate) [28,29]. Plant height has also been linked to the trade-off between stress tolerance and competitive ability, with tall species more likely to occur in productive habitats where light competition strongly influences community structure [25,26]. Thus, leaf traits and plant height reveal abiotic constraints on tree establishment. Both plant height and seed size (although seed size is often considered to affect dispersal, empirical evidence for this is inconsistent [30]) have been proposed as indicators of successional status in woody plants, with taller species and those with larger seeds being indicative of mid- to late-successional communities where light competition strongly influences community structure [25]. Thus, lower occurrence frequency of tall, large-seeded species in sites with low woody cover and distant from forest may reveal that early-successional colonizers are more able to establish at these sites.

The most accurate approach for quantifying dispersal ability is by estimating dispersal-distance probability curves using field observations or models [17]. However, accurate estimation of dispersal curves is not practicable for large numbers of species. Dispersal mode (e.g. wind, frugivory, water) is a coarse yet useful proxy for species’ dispersal abilities [31,32]. For example, species that are dispersed by animals have greater mean and maximum dispersal distances than species dispersed by other dispersal modes [32,30]. Examining the relationships between dispersal mode and factors limiting tree occurrences may reveal whether increased dispersal ability enhances colonization success.

Most functional trait studies encompassing large spatial scales have aimed primarily to document factors influencing trait variation, and have not considered in detail whether the observed patterns may have implications for ecological processes [33-36]. By testing for relationships between ecological strategy or dispersal traits and the primary factors limiting tree occurrences, we may be able to use functional traits to infer the ecological processes limiting tree species establishment over large spatial scales.

### Functional diversity and processes driving tree species occurrences

Functional diversity indices [37,38] are indicators of trait-based constraints on species occurrences in local communities [20,39]. Declining functional diversity indicates intensification of trait filtering (i.e. exclusion of species with traits that are poorly adapted to local conditions [20,40]). We can use functional diversity indices to assess the relative importance of trait filtering for ecological strategy or dispersal traits in limiting tree establishment. We would expect functional diversity for ecological strategy traits to decrease as abiotic constraints on tree establishment intensify (since only stress-tolerant species will be able to colonize). Functional diversity for height and seed size should decrease with decreasing woodiness and increasing distance from forest (since only early-successional species will be able to colonize). Functional diversity for dispersal traits should decrease as dispersal limitation increases (since only species with high dispersal ability will be able to colonize). We are aware of only one study examining variation in functional diversity of plant communities at very large spatial scales [41]. However, this study was concerned largely with comparing trait variation at very different spatial scales (globally, within major biomes and within communities), rather than examining shifts in trait filtering along environmental gradients. By contrast, studies of functional diversity in animal communities at the national and continental scale have revealed increasingly intense environmental filtering with increased abiotic stress and disturbance [39,42,43].

New Zealand has experienced a drastic reduction in forest cover since human arrival about ad 1200 [44,45]. Consequently, there are large areas of New Zealand currently covered by non-forest vegetation that could potentially support forest [46,47]. Large areas of marginal agricultural land have been recently abandoned or retired from production, providing great potential for afforestation by indigenous species [48].

The latitudinal range spanned by the main islands of New Zealand generates a large amount of climatic variation, with climate types ranging from subtropical in the northern North Island to sub-Antarctic on Stewart Island. Also, a main axial range runs almost the entire length of New Zealand, providing large areas of mountain and subalpine habitat. New Zealand also has a vast database of vegetation survey plots [49] spanning various environment types and landscape contexts (e.g. lesser or greater degrees of deforestation). This provides an excellent opportunity to understand the influence of abiotic constraints, local vegetation and proximity to seed sources on tree occurrence in non-woody vegetation. Finally, we have assembled a comprehensive database for key ecological strategy (potential height, leaf area and seed size) and dispersal (relating to dispersal adaptations and mode of dispersal) traits for all indigenous New Zealand tree species. This permits us to test whether trait data can improve our understanding of the processes driving afforestation.

### Aims and objectives

Our first aim was to identify the most important environmental and land cover variables influencing tree species occurrence in non-forest vegetation. We adapted the Kyoto Protocol definition of forest vegetation as having woody species greater than 5 m tall (termed tree species henceforth for simplicity) at high enough densities to potentially achieve 30% cover [50]. Since it is difficult to assess the potential for tree species to reach 30% cover we defined non-forest plots as having <30% cover of tree species. In an attempt to understand succession to Kyoto-defined forest, we modeled occurrence probability of tree species in non-forest plots. To this end we constructed predictive models of tree occurrence in non-forest survey plots including measures of the abiotic environment (e.g. temperature and water availability), local vegetation cover (i.e. vegetation type and local woodiness) and proximity to seed sources (i.e. distance to nearest forest and neighborhood forest cover) as predictor variables. We then used our functional trait data to test a suite of hypotheses on the processes by which abiotic environment, local vegetation cover and proximity to seed sources influence tree occurrence:

**H1:** Relative frequency of tall species and species with large leaves will decrease with abiotic stress.

**Reason:** Tall and large-leaved species are less tolerant of abiotic stress.

**H2:** Relative frequency of tall species and species with large leaves will decline with declining local woody cover.

**Reason:** Local woody cover reduces abiotic stress during tree establishment, favoring establishment of large-leaved species. Light competition increases with local woody cover, favoring tall species. Also, tall species are less able to persist following intense disturbance causing removal of all or most woody cover.

**H3:** Relative frequency of species with large seeds will decrease with declining local woody cover and proximity to seed source.

**Reason:** Species with large seeds will be poorly adapted to dispersing to sites further from forest. Successful establishment in non-woody vegetation is less likely to be influenced by shade tolerance (large seeds are associated with shade tolerance in the seedling phase).

**H4:** Relative frequency of species with traits enhancing dispersal and with multiple modes of dispersal will increase with declining proximity to seed source.

Reason: Only species with high dispersal ability are able to colonize sites distant from forest.

**H5:** Functional diversity for ecological strategy traits will decrease with abiotic stress and distance from seed source and increase with local woody cover.

**Reason:** Environmental filtering for ecological strategy will be stronger in more stressful environments and in sites further from seed sources. Woody cover reduces abiotic stress, and hence environmental filtering, during tree establishment.

**H6:** Functional diversity for dispersal traits will decrease with distance to seed source.

**Reason:** Only species with particular combinations of dispersal traits can colonize sites distant from seed source.

## Methods

### Vegetation survey dataset

New Zealand’s National Vegetation Survey Databank (NVS) contains records from approximately 77,000 vegetation survey plots encompassing forest, shrubland and non-woody vegetation [49] (http://www.givd.info/ID/AU-NZ-001). Plots using any one of three different data collection methods were considered for analysis. On the first plot type the abundance of each species present was recorded in seven fixed height tiers, using a modified Braun-Blanquet cover-abundance scale [51]. On the second type, the frequency of each species was recorded using 50 circular subplots, 15 cm in diameter, centered every 40 cm along a transect 20 m long [52]. On the third type, the frequency of each species was recorded using 50 quadrats (0.25 m^2^) centered every 2 m along a transect 100 m long [53]. In view of the potential for differences in survey methods to introduce bias we conducted our analyses separately on plots, using only one of the three methods. The main results obtained for the entire dataset were also obtained for each of the three survey methods separately. Furthermore, survey method was only a very weak predictor of tree occurrence probability. Consequently, it is highly unlikely that the main findings of our study can be explained by artifacts associated with differences in survey method.

**Figure 1:**
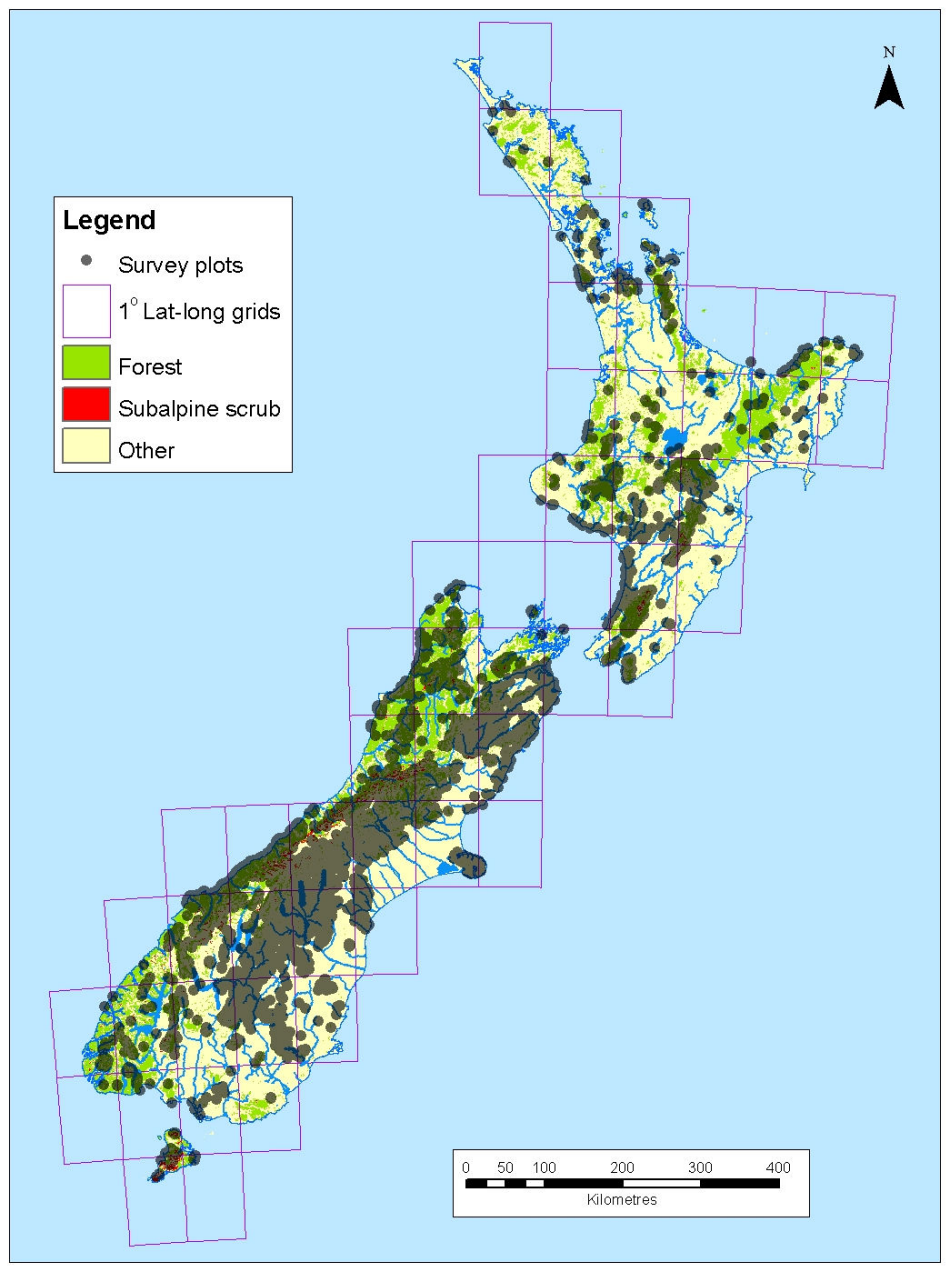
Map of survey plots used in boosted regression tree modeling. Vegetation classes for New Zealand survey plots are mapped based on a reclassification of Dymond and Shepherd [58]. ‘Other’ is all non-forest vegetation except subalpine scrub.

From these candidates, we selected 10,061 plots for our analysis (Figure 1). Criteria for inclusion were (1) the plot location was recorded with a precision of at least 100 m (2), if there were multiple plot records for a given location, only the most recent plot measurement was retained (3), plots were not related to exclosures or other experimental treatments (4), plots were located below 1400 m elevation to exclude plots that were definitely in alpine zones (5), woody species with potential height ≥ 5 m comprised <30% of the total cover or total frequency across the plot, to match the Kyoto Protocol definition of non-forest vegetation [50] (heights were taken from McGlone et al. [54]) (6), plot mean canopy height was ≤5 m. Each plot was scored as to whether any tree species were present (termed ‘tree occurrence’ hereafter). The tree species present in these plots may either be post-deforestation recruits or individuals that have survived deforestation. The criteria we used to select plots should exclude adult forest-dwelling trees, so that almost all of our occurrences will be contributed by new recruits or saplings that have survived deforestation. Plot surveys were conducted between 1982 and 2008. Over 80% of the plots were surveyed between 1982 and 1990, with around 10% surveyed between 1990 and 2000 and around 10% surveyed after 2000. These plot data are publicly available through the NVS website.

In most parts of New Zealand tree line naturally occurs below 1400 m, with mean annual temperature of 5.9°C generally defining the tree line [48]. Consequently, our dataset encompasses plots that naturally would not support forest (mainly due to climatic stress) and plots where forest has been removed by human or natural disturbance (mainly landslides associated with extreme rainfall or earthquakes). Eighty-seven per cent of plots have mean annual temperature of >5.9°C, based on climate maps summarizing over 30 years of temperature data [55] (Figure S1b). Consequently, extreme low temperatures will not be the sole reason for the absence of forest in most of our dataset. We deemed it advantageous to include some plots above tree line since this gives us more power to detect the effect of abiotic stress on tree species occurrences.

### Functional trait data

To determine seed mass, seeds of different species were sourced from the seed collection housed at the Allan Herbarium, Lincoln, New Zealand (CHR). For large-seeded species, mass was determined for five replicates of 10 seeds each; for small-seeded species, from five replicates of 50 seeds each. Replicates were placed in glass vials and dried overnight at 80°C. They were then placed in a desiccator and subsequently weighed on a Precisa 40SM-200A Weddenburn balance. Weights were recorded to the nearest 0.01 mg. Seed mass data are available from the Landcare Research ecological traits database (http://ecotraits.landcareresearch.co.nz)

We sourced data on tree height (m) and leaf size (mm^2^) from McGlone et al. [54]. Dispersal trait data were sourced from Thorsen et al. [56]. From this dataset we used four traits to describe dispersal: (1) nature of morphological dispersal adaption (none, aril, fleshy, hooked, minute, pappate, viscid or winged); (2) principal dispersal mode (ballistic, ‘mobile’ – i.e. epizoochorous, by frugivory, water or wind); (3) number of dispersal modes; and (4) whether or not the species is biotically dispersed. There was no evidence of strong correlations between either ecological strategy or dispersal traits (all pairwise Pearson correlations <0.5). Height and dispersal data are available from published appendices linked to McGlone et al. [54] and Thorsen et al. [56] respectively.

### Environmental and land cover predictor data

We used a variety of complementary environmental and land cover variables to predict tree occurrence. The climatic variables were taken from the GIS climate surfaces described by Leathwick et al. [55]. The variables we used were: mean and minimum annual temperature (°C), mean annual rainfall (mm), mean annual solar radiation (kJ.day^-1^.m^–2^), October soil water deficit and vapor pressure deficit (kPa), mean wind speed for the month of October (when prevailing westerly winds are most active, km.h^-1^), soil particle size, phosphorus, and drainage (all ordinal rankings). Our land cover variables were derived from either Land Cover Database 2 (LCDB2 [57]; http://koordinates.com/layer/1072-land-cover-database-version-2-lcdb2/) or Ecosat [58]. LCDB2 documents aerial extent estimates of 61 classes of land cover or land use derived from a classification of Landsat satellite imagery acquired in the austral summers of 1996/7 and 2001/2. Ecosat used satellite imagery as well to produce a land cover classification at a 1:50,000 scale. This classification reflected the proportions of *Nothofagus* , other broadleaved species, and conifer species in the forest, as each has a unique spectral signature. The approach was then used to derive a national woody vegetation layer comprising nine classes. We used reclassified LCDB2 cover classes (herbaceous and bare ground, shrubland, planted forest, broadleaved forest and indigenous forest, following Mason et al. [59]) as a measure of vegetation type in the immediate vicinity of the plot. We derived four variables from Ecosat: distance to nearest forest, percentage forest cover within a radius of 100 m, percentage local woody cover (either shrubland or forest) within a radius of 25 m, and the dominant vegetation type within a radius of 100 m. Distance to nearest forest gives an indication of proximity to seed sources, while percentage forest cover within a 100 m radius gives a more fine scale (< 100 m) measure of seed source availability in the local neighborhood. Percentage woody cover within a 25 m radius quantifies woodiness in the immediate vicinity of the plot, while the dominant Ecosat vegetation type within 100 m indicates neighborhood vegetation type, and provides more detail on the variation in forest composition than does LCDB2. Figure S1 presents histograms and bar charts showing plot frequency distributions with respect to the predictor variables. All environmental and land cover data were taken from GIS grids with 100m x 100m pixel size, so that the values assigned to each plot were those for the 100m grid square in which it occurred. Percentage woody cover within a 25 m radius was converted to 100m coverage by applying the value of the 25-m pixel at the top left corner of the 100-m pixel to the whole 100-m pixel. This is a very common practice in GIS analyses, because different types of variables – climate, land cover, topography – are typically mapped at different scales. Obtaining values for each variable at each plot location requires that they all be mapped at the same resolution. All GIS data are publicly available on request to the corresponding author.

Some care must be exercised around our use of local woody cover and local vegetation type to predict tree occurrence. Many of our plots were surveyed prior to the remote sensing used in Ecosat and LCDB2 (the land cover classifications we used). Consequently, there is a risk of circularity since local woodiness might be influenced by the presence of tree species in our plots – with plots containing tree species perhaps more likely to have high woody cover due to the presence of tree species. Despite this, our plot selection criteria mean that the woody cover is largely contributed by shrubs or arborescent species (i.e. woody species with maximum height < 5 m). Disturbance disrupts the relationship between tree species distribution and environmental variables in many regions of the globe (e.g. [60]), with this effect being particularly strong when humans are the primary disturbance agent (e.g. [59]). Consequently, data on vegetation in the immediate vicinity of the plots are required to improve our ability to predict tree species occurrence, even though this might risk the type of circularity described above. Indeed, when we excluded local land cover variables from our set of predictor variables, predictive accuracy decreased by >10%. Examining the relationship between local vegetation cover and functional traits of tree species may be helpful by providing a test for non-circular ecological mechanisms by which local vegetation cover influences tree occurrence probability.

### Predictive modeling of tree occurrence probability

We used boosted regression tree (BRT) modeling [61] to predict the probability of at least one tree species occurring in survey plots (‘tree occurrence probability’ henceforth) based on our environmental and land cover variables. We chose to use BRTs as they perform well in comparison to competing methods [61] and model interactions between predictors very effectively, without requiring explicit interaction terms to be defined *a priori*. In all BRT models we used a tree complexity of 5 and learning rate of 0.001. The maximum number of trees was set at 20,000, though this limit was never reached. Model goodness of fit was assessed using the cross-validated area under the relative operating characteristic (ROC) curve [62], which is a standard measure of how well a model discriminates between presences and absences. Cross validation was performed by randomly removing 10% of the plots from the dataset, fitting the BRT model on the remaining plots and then assessing the goodness of fit on the removed plots. In machine learning methods like BRT, the use of cross validation in assessing goodness of fit is vital to prevent over-fitting. To further reduce the chance of over-fitting, we conducted a step-wise model simplification process that sequentially removes the predictor variable whose removal causes the smallest decrease in goodness of fit. We performed this model simplification using the gbm.simplify function implemented in R [63] provided in the supplementary material of Elith et al. [61]. Following the simplification procedure we fitted a new model containing only the variables whose removal caused a non-negligible decrease in goodness of fit. Analyses were performed in R version 2.13.

### Testing relationships between traits, and environment and land cover

We used the fourth-corner approach [64,65] to test the significance of relationships between functional traits and the best predictor variable from each of the three main types of predictor: land cover, proximity to seed source and abiotic stress. Specifically, we assessed significance by combining results from Dray and Legendre’s [64] model 2 (which randomizes entire rows - i.e. sites – of the site x species table) and model 4 (which randomizes entire columns - i.e. species – of the site x species table). In this way, the models respectively break the links between sites and predictor variables and between species and traits. To control for variation in regional species pools throughout our study area, we restricted the randomization in model 2 so that only plots on the same island and within the same 1° grid square were swapped. Significance (at α = 0.05) for the relationship between each trait and predictor variables was assessed using the test (i.e. either model 2 or model 4) that gave the largest *p*-value. Dray and Legendre had initially suggested using α = 0.05^1/2^, but ter Braak et al. [65] demonstrated that α = 0.05 gives appropriate error rates. For quantitative traits we used the Pearson correlation coefficient as the test statistic. For categorical traits, we used the Pearson correlation between each individual category and the predictor variables. Analyses were performed in R version 2.13.

There was no evidence of strong colinearity between the environmental variables used in our trait analyses (all correlations had Pearson *r* < 0.5). Nor were the ecological strategy traits strongly correlated with each other or with the dispersal traits we used. For these reasons the RLQ analyses (a multivariate extension of the fourth-corner approach [66]) we attempted did not yield any readily interpretable ordination axes for either the functional traits or site variables that we analyzed. This result, combined with the lack of colinearity between site variables and between functional traits, justifies our use of the fourth-corner approach for examining relationships between traits and site variables.

### Functional diversity relationships with environment and land cover

We quantified functional diversity using Rao quadratic entropy [67]. We used species presence/absence as our abundance measure, with present species assigned an abundance of 1 and absent species an abundance of 0. Rao calculated using presence/absence is largely a measure of functional richness (i.e. the volume of niche space occupied by the species [68]). We calculated Rao separately for each of the ecological strategy traits. We calculated a single multivariate measure of Rao for dispersal traits, using all of our dispersal traits to estimate distances between species in dispersal trait space. We chose not to use the more standard functional richness measure (convex hull volume [69]) because it is only interpretable if the number of trait dimensions is less than local species richness. This would have posed a problem for analyzing the functional diversity of dispersal traits, where multiple traits were analyzed simultaneously. In all cases, Gower’s distance was used to estimate dissimilarity between all species pairs, since this permits dissimilarity to be estimated using both continuous and categorical variables [70].

Functional richness is often positively correlated with species richness even in the absence of any ecological processes (i.e. with randomly generated data [68]). To control for species-richness effects, observed Rao values must be compared with those expected under a null model. We used a matrix-swap null model [71], which randomizes species co-occurrences while maintaining species occurrence frequencies and plot species richness to be the same as in the observed data. To control for variation in regional species pools we restricted randomizations by island and within grid squares of 1° latitude and longitude, so that species occurrences were only randomized between plots on the same island and within the same 1° grid square (using a restricted randomization approach [39]). We expressed observed Rao values for each plot using the standardized effect size (SES) of Gotelli and McCabe [72].

## Results

### Spatial models of tree occurrence

**Figure 2:**
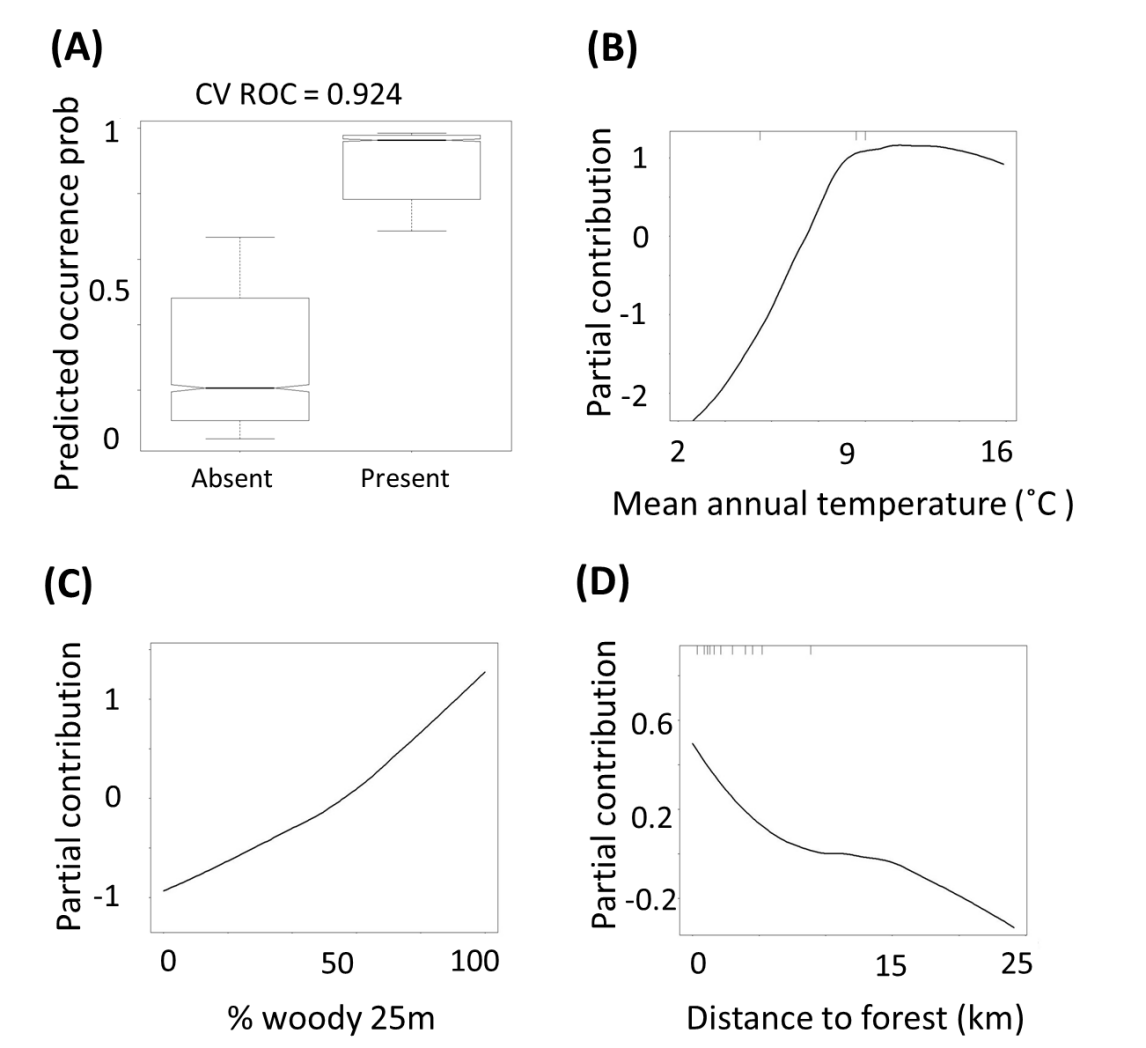
Partial contributions to Observed vs. Fitted tree occurrences within the simplified BRT model. The graphs show Observed vs. Fitted tree occurrences (A) and smoothed partial contributions within the simplified BRT model for (B) mean annual temperature (C) percentage woody cover in a 25 m radius and (D) distance to nearest forest. The smoothed partial contribution plots reflect the influence of a predictor variable when all other variables are held constant. CVROC is the cross-validated receiver operator curve (ROC) for the final boosted regression tree model. ROC is a measure of discrimination accuracy when predicting a binary response.

**Figure 3:**
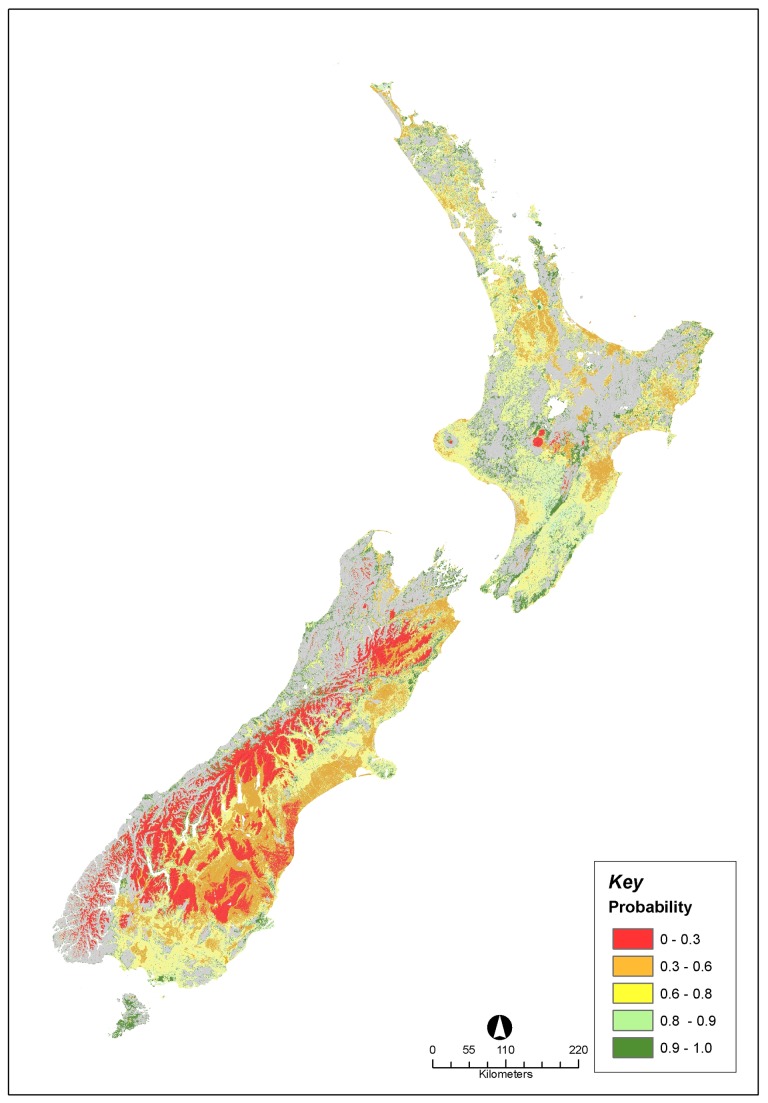
Map of predicted tree occurrence probability in non-forest vegetation in New Zealand. The map shows model-predicted probability of tree occurrence in non-forest vegetation (the ‘other’ and ‘subalpine scrub’ classes of Dymond and Shepherd [58]) in New Zealand. Grey areas are covered by indigenous (from [58]) and planted forest (from Land Cover Database 2).

The BRT model of tree occurrence probability predicted tree occurrences very accurately (cross-validated ROC = 0.924, Figure 2). Figure 3 presents a map of predicted tree occurrence probability for all non-forested areas of New Zealand. Mean annual temperature and local woody cover were the two most influential predictors in the simplified BRT model of tree occurrence (Figure 2, Table 1). The relationship for mean annual temperature was asymptotic, with minimal change in predicted tree occurrence probability above 9°C. The relationship for local woody cover was positive and monotonic, with predicted tree occurrence probability increasing with increasing woody cover. The influence of almost all other variables in the model was an order of magnitude lower than for mean annual temperature and local woody cover (Table 1, Figure S2). Amongst these secondary predictors, notable relationships were a monotonic increase in predicted occurrence with minimum temperature and rainfall and a monotonic decline with distance from nearest forest.

**Table 1:** Relative contribution of predictor variables in the simplified boosted regression tree model predicting tree species occurrence probability.

**Variable**	**Contribution**
Mean annual temperature	44.9
% woody cover within 25 m	34.1
Mean annual rainfall	5.0
Mean annual solar radiation	3.9
Minimum annual temperature	3.5
Distance to forest	2.5
October soil water deficit	2.3
Mean October wind speed	2.2

‘Contribution’ is the percentage of splits in the data made using each variable – regression trees are fitted by sequential dichotomous divisions of the data.

The two most important interactions were between mean annual temperature and local woody cover (contribution 12.5%, Figure S3), and between minimum temperature and distance to nearest forest (contribution 5%, Figure S4). Occurrence probability at low mean annual temperature was higher at high levels of local woody cover. This might be due to woody cover reducing the negative effects of low mean annual temperature on tree occurrence probability. For the minimum temperature – distance to forest interaction, occurrence probability at low minimum temperature was higher when distance to forest was lower and vice versa. This suggests that higher minimum temperatures counteracted the effects of dispersal limitation (by increasing establishment probability of seeds dispersing to sites distant from forest, and hence compensating for lower propagule pressure). It also indicates that proximity to seed source counteracted the negative effects of low temperature on tree occurrence probability (by increasing propagule pressure, and hence compensating for the lower per-individual establishment probability).

### Trait relationships with environment and land cover

Of the ecological strategy traits only height was strongly related to both environmental and land cover variables (Table 2). In general, the relative frequency of short species was greater in colder plots or plots further from forest and with less woody cover locally. The relative frequency of small-seeded species increased with mean temperature, but was not related to land cover variables or distance to nearest forest. Dispersal traits, except type of dispersal adaptation, were strongly influenced by land cover. Specifically, the relative frequency of biotically dispersed species, species with only a single mode of dispersal, and species primarily dispersed by frugivory was higher in plots with high local woody cover and close to forest. The opposite pattern was observed for species with no dispersal adaptations and those principally dispersed by ballistic means. Dispersal traits were also significantly related to mean annual temperature. In general, biotically dispersed species, species with only a single mode of dispersal and those principally dispersed via frugivory, and those with morphological adaptations for frugivory such as arils or fleshy fruits, were more likely to occur in warmer plots. Species lacking any morphological dispersal adaptations tended to occur in colder plots.

**Table 2:** Correlations between ecological strategy and dispersal trait and key land cover and environmental variables from fourth-corner analyses.

		**Mean temp.**	**Distance to forest**	**% woody 25 m**
**Ecological strategy**	Tree height (m)	**0.126**	**−0.155**	**0.158**
	Leaf size (mm^2^)	0.055	−0.084	**0.104**
	Seed mass (mg)	**0.128**	−0.054	*0.061*
**Dispersal adaptation**	None	−0.065	**0.281**	**−0.299**
	Aril	*0.098*	−0.039	0.060
	Fleshy	*0.099*	−0.122	*0.134*
	Hooked	−0.062	**−0.063**	*0.046*
	Minute	**−0.144**	0.048	−0.048
	Papate	−0.022	−0.028	0.014
	Viscid	0.032	0.073	−0.033
	Winged	**−0.124**	−0.047	0.027
**Principal dispersal mode**	Ballistic	**−0.180**	**0.387**	**−0.488**
	Frugivory	**0.186**	**−0.149**	**0.185**
	Mobile	0.027	0.026	−0.013
	Water	0.040	0.024	−0.024
	Wind	**−0.133**	0.018	−0.021
**Number of dispersal modes**	1	**0.202**	**−0.208**	**0.265**
	>1	**−0.177**	**0.221**	**−0.284**
**Biotically dispersed?**	Biotic	*0.093*	**−0.180**	**0.194**

The correlations are between species occurrences and predictor variables, so that a positive correlation for continuous ecological strategy traits indicates that species with a high trait value are more likely to occur in sites with a high value for the predictor variable. For example, the positive correlation between height and mean annual temperature indicates that tall species are more likely to occur in warmer plots. For categorical dispersal traits, a positive correlation indicates that species possessing the trait in question are more likely to occur in plots with a high value for the predictor variable. Values highlighted in bold indicate significance at *p* < 0.05; values highlighted in italics indicate significance at *p* < 0.1. Significance was assessed following ter Braak et al. [65], using null models where either whole columns (species) or rows (sites) of the species × site table were randomized.

### Functional diversity relationships with environment and land cover

Functional diversity (relative to random expectation) for all of the ecological strategy traits was positively correlated with mean temperature and local woodiness and was negatively correlated with distance to forest (Table 3). By contrast, functional diversity for dispersal traits was negatively correlated with mean temperature and local woodiness and was positively correlated with distance to forest. This shows that the factors limiting tree occurrence (low temperature, low woody cover and high distance from forest) also filtered out tree species based on ecological strategy. By contrast, it seems that these factors did not at all filter out tree species based on their dispersal traits. Rather the functional diversity of dispersal traits was higher in plots where tree occurrence was more marginal. This demonstrates that tree species have developed a variety of solutions to the problem of dispersing into non-woody vegetation.

**Table 3:** Correlations between functional diversity and key land cover and environmental variables.

	**Mean temp.**	**Distance to forest**	**% woody 25 m**
Tree height (m)	0.332	−0.303	0.247
Leaf size (mm^2^)	0.087	−0.158	0.187
Seed mass (mg)	0.330	−0.148	0.145
Dispersal adaptation	−0.414	0.223	−0.261

Functional diversity is Rao quadratic entropy expressed relative to null expectation.

All results are significant at *p* < 0.05.

## Discussion

Our results show that it is possible to use a set of basic environmental and land cover variables to accurately predict tree species occurrence (i.e. the presence of at least one tree species) in non-forest vegetation over very large spatial scales. We also found strong evidence for relationships between trait values, functional trait diversity and key predictors of tree species occurrence. Table 4 summarizes the relevance of these results for the hypotheses we posed in the introduction. These revealed that abiotic stress (particularly low temperatures), local woody cover and proximity to seed sources interacted with traits to influence tree occurrence probability. In particular, they demonstrate that as temperature and woody cover decrease and distance from seed source increases, trait filtering intensifies for ecological strategy traits but not dispersal traits.

**Table 4:** Relevance of trait analyses for hypotheses.

**Hypothesis**	**Reject/Accept**	**Evidence**	**Conclusion**
**H1**: Relative frequency of tall species and those with species with large leaves will decrease with abiotic stress.	True for height, not for leaf size	Table 2	Tall species are less tolerant of low temperature stress but there was no evidence of this for large-leaved species.
**H2**: Relative frequency of tall species and species with large leaves will decline with declining local woody cover.	True for both leaf size and height	Table 2	Tall species favored by increased light competition with greater woody cover. Woody cover reduces abiotic stress and provides protection from herbivores, which favors large-leaved species.
**H3**: Relative frequency of species with large seeds will decrease with declining local woody cover and distance to seed source.	False	Table 2	Seed size does not influence dispersal ability or ability to establish in non-woody vegetation.
**H4**: Relative frequency of species with adaptations enhancing dispersal and with multiple modes of dispersal will increase with declining proximity to seed source.	True for multiple modes of dispersal, not true for dispersal adaptations	Table 2	Having multiple modes of dispersal increases ability to disperse into non-woody habitats distant from seed sources, but having specialized dispersal adaptations does not.
**H5**: Functional diversity for ecological strategy traits will decrease with abiotic stress and distance from seed source and increase with local woody cover.	True	Table 3	The range of viable ecological strategies for tree species is lower in non-woody vegetation and further away from seed sources.
**H6**: Functional diversity for dispersal traits will decrease with distance to seed source.	False	Table 3	Tree species establishing in non-woody vegetation or in vegetation distant from seed sources employ a wide range of dispersal strategies.

‘Reject/Accept’ indicates whether the hypothesis was rejected or accepted in the basis of our analyses and ‘Evidence’ indicates where the evidence for rejecting or accepting a hypothesis is presented.

### Trait values and abiotic predictors of tree occurrence

Relative frequency of tall tree species and species with large seeds decreased with declining temperature. Since we used a geographically constrained null model to assess the significance of trait correlations, the effect of temperature is unlikely to be due to latitudinal variation in the proportion of tall, large-seeded species in regional species pools. Rather, the effect of temperature on height and seed size is likely due to altitudinal variation in temperature. These results most likely reflect a decline in the influence of light competition on fitness as stress from low temperatures intensifies [25]. Growing taller than competitors provides the obvious advantage of pre-empting their light capture, so that superior height represents a strong fitness advantage when light competition is intense. Large seed size enhances the shade tolerance of seedlings, and is a characteristic of many New Zealand tree species adapted to regenerate beneath mature forest canopies (e.g. *Beilschmiedia* *tawa* Lauraceae, and *Prumnopitys* *ferruginea* Podocarpaceae). Declines in height and seed size with declining temperature reflect a decline in the fitness advantage gained from these strategies due to reduced light competition. Temperature is a key determinant of forest biomass and canopy height in New Zealand, with both declining markedly below a mean annual temperature of 9°C [59]. This supports the interpretation of declining height and seed size as indicative of a decrease in light competition with declining temperature.

The main trends for dispersal traits were a decline in frequency for biotically dispersed species with declining temperature (particularly those species with adaptations to enhance frugivory). By contrast, frequency of species with more than one mode of dispersal and species without any dispersal adaptations increased with declining temperature. Thus, in cooler areas dispersal by animals played a relatively minor role in tree establishment. Once again, because we used a geographically constrained null model, the observed effect of temperature on dispersal traits is unlikely to be due to latitudinal variation in the frequency of dispersal traits in regional species pools.

### Trait values and land cover predictors

Tall tree species increased in frequency with increasing local woody cover and neighborhood forest cover and declined with increased distance from forest. This perhaps reflects the trade-off between colonization and competition [73], with tall, late-successional species adapted for intense light competition being less able to colonize non-woody vegetation or sites distant from forest than short, early-successional species. In the New Zealand flora, only one of the common pioneer tree species ( *Kunzea* *ericoides* Myrtaceae) grows taller than 10 m [54]. Thus, the results for tree height suggest that the land cover variables we used do successfully capture barriers to colonization by mid- to late-successional tree species.

Species with large leaves increased in frequency with increasing local woody cover, indicating that woody cover may have facilitated establishment of stress-intolerant, large-leaved species by reducing abiotic stress. Indeed, the strong interaction effect between mean annual temperature and local woody cover suggests that increasing woody cover reduced the negative effect of low temperatures on tree occurrence probability. The positive relationship between leaf area and woody cover may also arise from woody cover providing a refuge from herbivores, since experimental evidence has shown that leaf area is a key trait in selection of New Zealand tree species by ungulate herbivores [74]. Mason et al. [4] found that tree species with foliar traits typical of high stress tolerance and low palatability (i.e. low nitrogen, low specific leaf area and high lignin content) dominated the earliest stages of a New Zealand post-pastoral succession. Our results for leaf area show that this pattern of grassland colonization by stress-tolerant, unpalatable trees may apply throughout New Zealand.

Dispersal is a highly stochastic process, and this stochasticity often makes it difficult to detect dispersal patterns. The fact that we obtained such clear evidence for the influence of dispersal limitation on traits emphasizes that large datasets, such as the one used in this study, may overcome the stochasticity caused by rare, random dispersal events, such as long distance dispersal, to reveal dispersal-driven patterns [75]. Relative frequency of biotically dispersed species, particularly those dispersed primarily by frugivory, increased with local woodiness and decreased with distance to forest. By contrast, frequency of abiotically-dispersed species, species with no obvious dispersal adaptations and more than one mode of dispersal decreased with local woodiness and increased with distance to forest. Thus, there was no evidence for vertebrate dispersal enhancing establishment in non-woody vegetation or in sites distant from forest. Numerous studies in forest–savannah mosaics have found that woody cover enhanced colonization of vertebrate-dispersed species into disturbance-induced non-forest vegetation [14-16]. This reveals a strong tendency for vegetation cover to interact with disperser behavior. In the New Zealand context this especially relates to woody species providing perches for frugivorous birds [13]. At least one other study [76] also found that greater distances from mature forest reduced the percentage of biotically dispersed species at sites in tropical and subtropical forests, but this pattern did not hold in temperate forests. Our results suggest that, for tree species, having seeds with singular morphological adaptations (e.g. flesh or wings) does not enhance their ability to establish in non-woody vegetation, or in sites distant from seed sources. Instead, tree species with a ‘jack of all trades, master of none’ approach to dispersal (multiple modes) are better at establishing in non-woody vegetation or in sites far from seed sources.

### Functional diversity, abiotic stress and land cover

Functional diversity revealed that environmental filtering for ecological strategy traits intensified with declining temperature, decreasing woodiness and increasing distance from forest. Thus, more intense environmental filtering for ecological strategy followed the same pattern as tree species occurrence probability. This shows that the factors limiting tree species occurrence – low temperatures, low local woody cover and distance to seed source – did so by restricting the range of viable ecological strategies. There is a growing body of evidence that functional diversity in plant communities decreases with increasing abiotic stress, particularly for traits linked to light capture strategy (e.g. [20,69]). However, we are unaware of any work demonstrating that functional diversity declines with dispersal limitation (i.e. distance from seed source).

By contrast, there was no evidence that low probability of tree species occurrence was associated with filtering for dispersal traits. Rather, functional diversity for dispersal traits was positively correlated with distance to forest and negatively correlated with temperature and local woody cover, whereas occurrence probability showed the opposite pattern. These results suggest that New Zealand tree species have found a variety of solutions to the problem of long-distance dispersal into non-woody vegetation. Grime [77] argues that trait filtering will be stronger for traits linked to resource-use strategy than for those linked to reproductive strategy. However, he did not consider the possibility that the intensity of filtering could vary along ecological gradients. Our results suggest that when trait filtering for ecological strategy is strongest, filtering for dispersal strategy is weakest.

### Functional traits improve ecological interpretability of predictive models

It may often be difficult to directly infer ecological processes influencing tree species colonization of non-forest vegetation from occurrences in ‘snapshot’ survey data. Firstly, observed tree species occurrences at a single point in time might arise either through persistence of individuals present before deforestation or through colonization following deforestation. Further, modeling tree species occurrences in non-forest vegetation requires estimators of local woody cover, which could potentially introduce circularity (since tree species may influence estimators of woody cover by their presence). Our results show how documenting relationships between functional trait values and diversity and the factors influencing tree species occurrences may help to overcome these problems and reveal potential ecological processes driving tree species colonization. In particular, our trait analyses show how the measures of abiotic stress, proximity to seed source and local vegetation cover most strongly influencing tree species occurrences might also limit dispersal and establishment of tree species in non-forest vegetation.

## Conclusions

Our results show how ‘snapshot’ survey plot data, combined with functional trait data, may reveal the processes driving tree species establishment in non-forest vegetation over large spatial scales. Our findings also reveal methods by which tree species establishment could be enhanced. For instance, tree establishment, especially for bird-dispersed mid- to late-successional species, will be aided by efforts to facilitate shrub invasion of grassland. Further, colonization of sites distant from existing forest by mid- to late-successional species capable of forming tall, high-carbon forests may require enrichment planting. This information has the potential to enhance attempts to sequester carbon through indigenous afforestation. It may also prove beneficial for ecological restoration planning, especially regarding spatial allocation of restoration planting to maximize carbon and biodiversity gain (e.g. [78]).

## Supporting Information

Figure S1 **Histograms and bar graphs showing distribution of plots with respects to the predictor variables used in boosted regression tree models of tree occurrence.** Bar graphs are used for categorical variables and are indicated by shaded columns. Histograms were used for continuous and ordinal variables. Data sources for each variable are provided in the methods section of the main document. Names for Forest type codes are: 1, Subalpine scrub; 3, Kauri forest; 4, Podocarp forest; 5, Podocarp–broadleaved forest; 6, Beech forest; 7, Broadleaved forest; 8, Podocarp–broadleaved/beech forest; 9, Beech/broadleaved forest; 10, Beech/podocarp-broadleaved forest; 12, Unspecified indigenous forest (mainly regenerating indigenous forest); 14, Other (all non-forest vegetation types except Subalpine scrub). Reclassified LCDB2 classes are: 1, Deciduous hardwood forest; 2, Herbaceous vegetation and bare ground such as beaches and river gravels; 3, Indigenous forest; 4, Planted forest (mainly exotic conifers); 5, Shrubland.
(PDF)

Figure S2 **Smoothed partial contributions of predictor variables to the final boosted regression tree model for tree species occurrence probability in non-forest plots.**
(PDF)

Figure S3 **Perspective plot of predicted tree occurrence probability (fitted value) in final boosted regression tree model against local woody cover (% woody cover in 25 m radius) and mean annual temperature.**
(PDF)

Figure S4 **Perspective plot of predicted tree occurrence probability (fitted value) in final boosted regression tree model against distance to forest and minimum temperature.**
(PDF)
