# Supplementary material for: Functional Traits Reveal Processes Driving Natural Afforestation at Large Spatial Scales
Source: PLoS One. 2013 Sep 18;8(9):e75219. doi: 10.1371/journal.pone.0075219 (PMC3776731; doi:10.1371/journal.pone.0075219)
Supplement: Figure S1 — Histograms and bar graphs showing distribution of plots with respects to the predictor variables used in boosted regression tree models of tree occurrence. Bar graphs are used for categorical variables and are indicated by shaded columns. Histograms were used for continuous and ordinal variables. Data sources for each variable are provided in the methods section of the main document. Names for Forest type codes are: 1, Subalpine scrub; 3, Kauri forest; 4, Podocarp forest; 5, Podocarp–broadleaved forest; 6, Beech forest; 7, Broadleaved forest; 8, Podocarp–broadleaved/beech forest; 9, Beech/broadleaved forest; 10, Beech/podocarp-broadleaved forest; 12, Unspecified indigenous forest (mainly regenerating indigenous forest); 14, Other (all non-forest vegetation types except Subalpine scrub). Reclassified LCDB2 classes are: 1, Deciduous hardwood forest; 2, Herbaceous vegetation and bare ground such as beaches and river gravels; 3, Indigenous forest; 4, Planted forest (mainly exotic conifers); 5, Shrubland. (PDF) [file pone.0075219.s001.pdf]

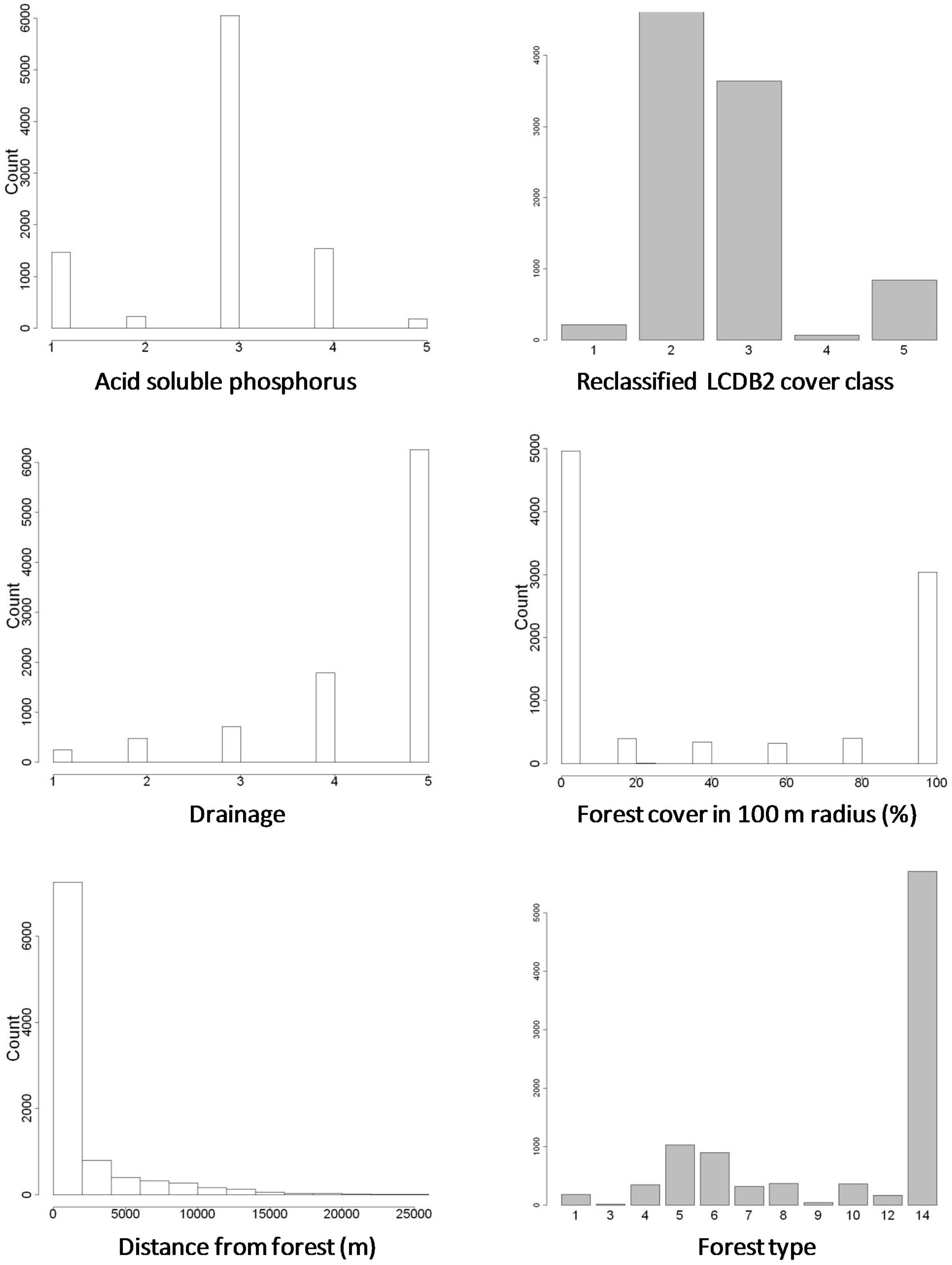
Count
Acid soluble phosphorus
Reclassified LCDB2 cover class
Count
Drainage
Count
Forest cover in 100 m radius (%)
Count
Distance from forest (m)
Forest type

**Figure S1a:**

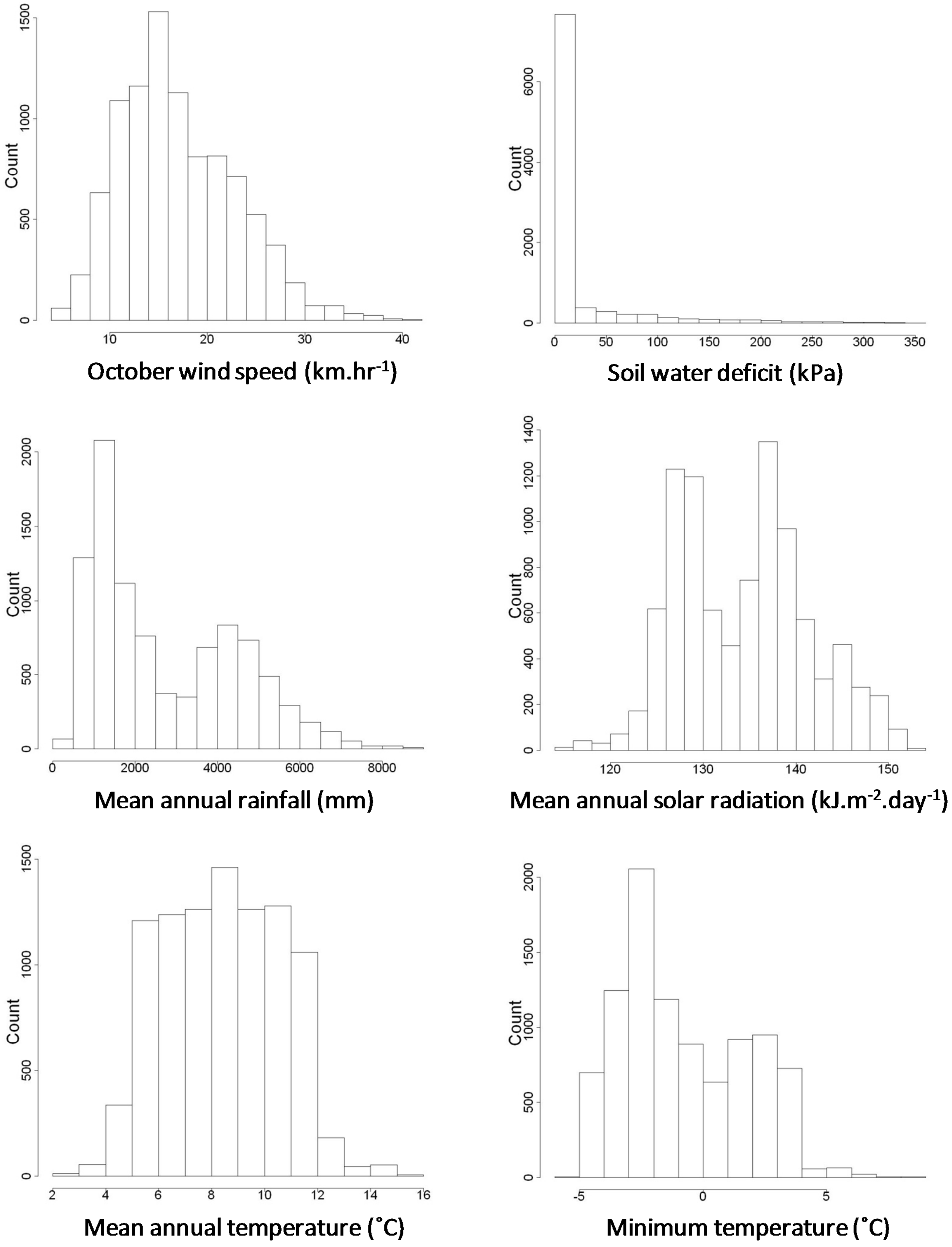
Count
October wind speed (km.hr-1)
Count
Soil water deficit (kPa)
Count
Mean annual rainfall (mm)
Count
Mean annual solar radiation (kJ.m-2.day-1)
Count
Mean annual temperature (˚C)
Count
Minimum temperature (˚C)

**Figure S1b:**

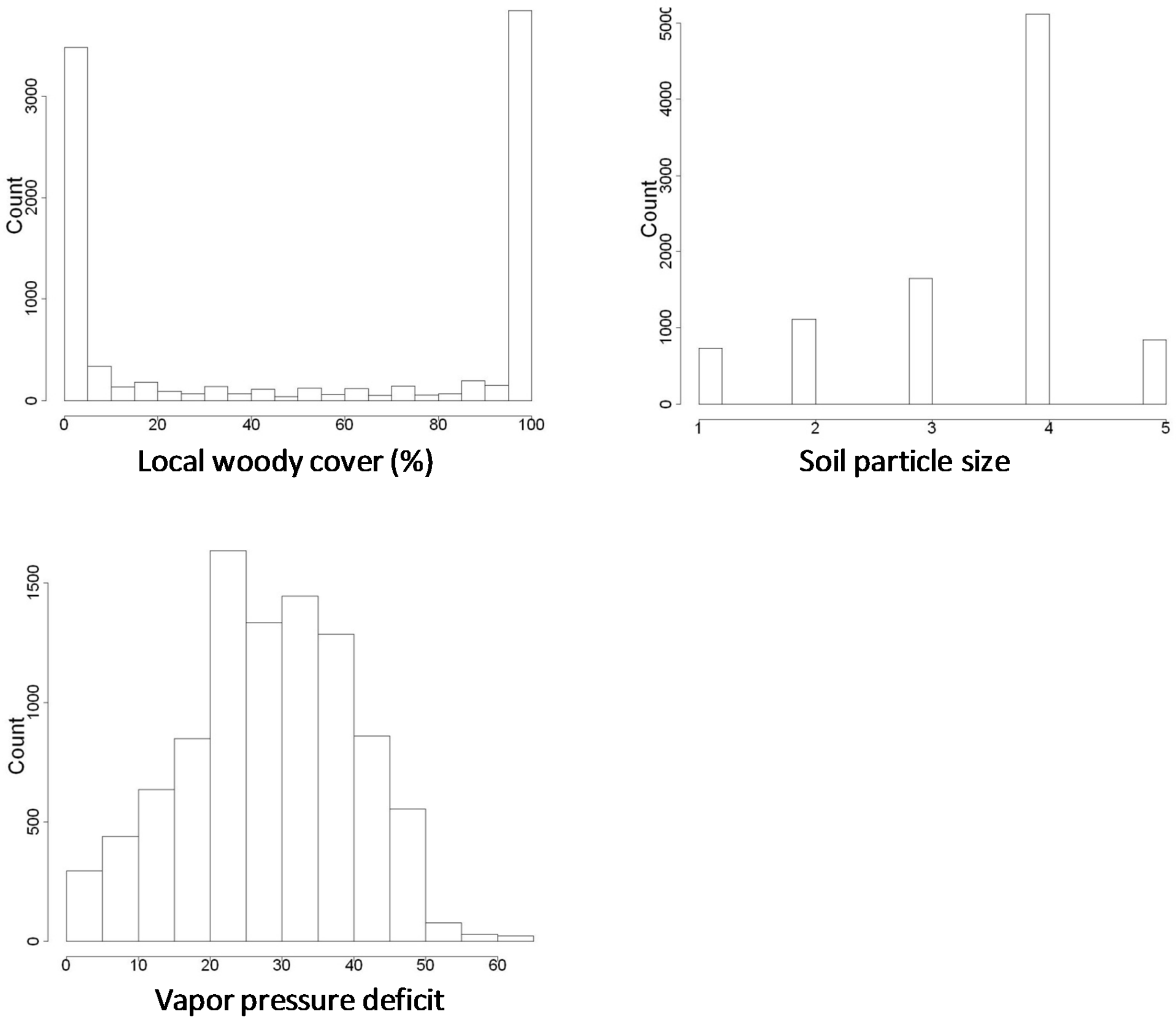


**Figure S1c:**

**Figure S1 caption:** Histograms and bar graphs showing distribution of plots with respects to the predictor variables used in boosted regression tree models of tree establishment. Bar graphs are used for categorical variables and are indicated by shaded columns. Histograms were used for continuous and ordinal variables. Data sources for each variable are provided in the methods section of the main document. Names for Forest type codes are: 1, Subalpine scrub; 3, Kauri forest; 4, Podocarp forest; 5, Podocarp-broadleaved forest; 6, Beech forest; 7, Broadleaved forest; 8, Podocarp-broadleaved/beech forest; 9, Beech / broadleaved forest; 10, Beech / podocarp-broadleaved forest; 12, Unspecified indigenous forest (mainly regenerating indigenous forest); 14, Other (all non-forest vegetation types except Subalpine scrub). Reclassified LCDB2 classes are: 1, Deciduous hard wood forest; 2, Herbaceous vegetation and bare ground such as beaches and river gravels; 3, Indigenous forest; 4, Planted forest (mainly exotic conifers); 5, shrubland.
