# Supplementary material for: Functional Traits Reveal Processes Driving Natural Afforestation at Large Spatial Scales
Source: PLoS One. 2013 Sep 18;8(9):e75219. doi: 10.1371/journal.pone.0075219 (PMC3776731; doi:10.1371/journal.pone.0075219)
Supplement: Figure S2 — Smoothed partial contributions of predictor variables to the final boosted regression tree model for tree species occurrence probability in non-forest plots. (PDF) [file pone.0075219.s002.pdf]

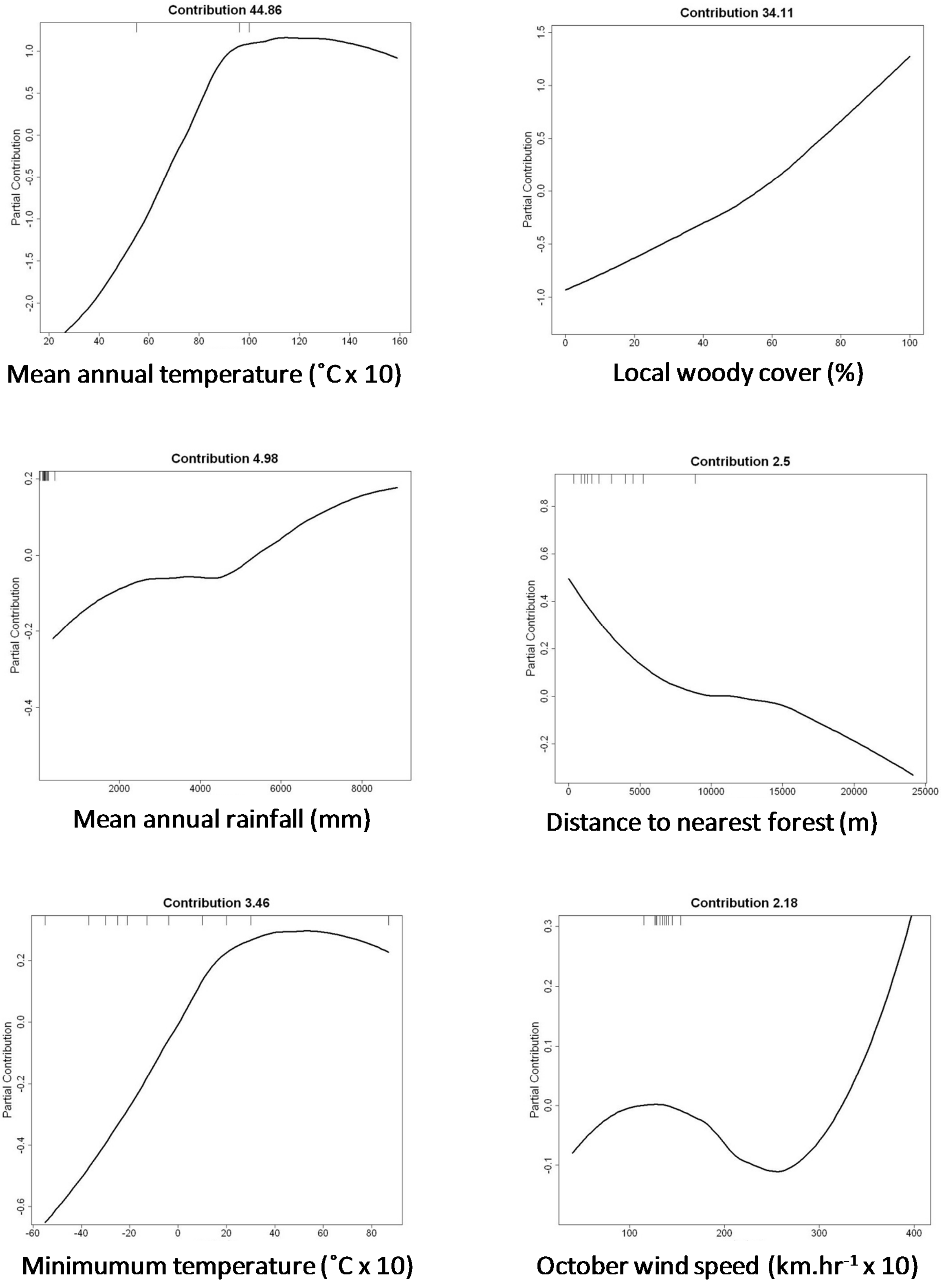
Contribution 44.86
Partial Contribution
Mean annual temperature (˚C x 10)
Contribution 34.11
Partial Contribution
Local woody cover (%)
Contribution 4.98
Partial Contribution
Mean annual rainfall (mm)
Contribution 2.5
Partial Contribution
Distance to nearest forest (m)
Contribution 3.46
Partial Contribution
Minimumum temperature (˚C x 10)
Contribution 2.18
Partial Contribution
October wind speed (km.hr-1 x 10)

Fig S2a:

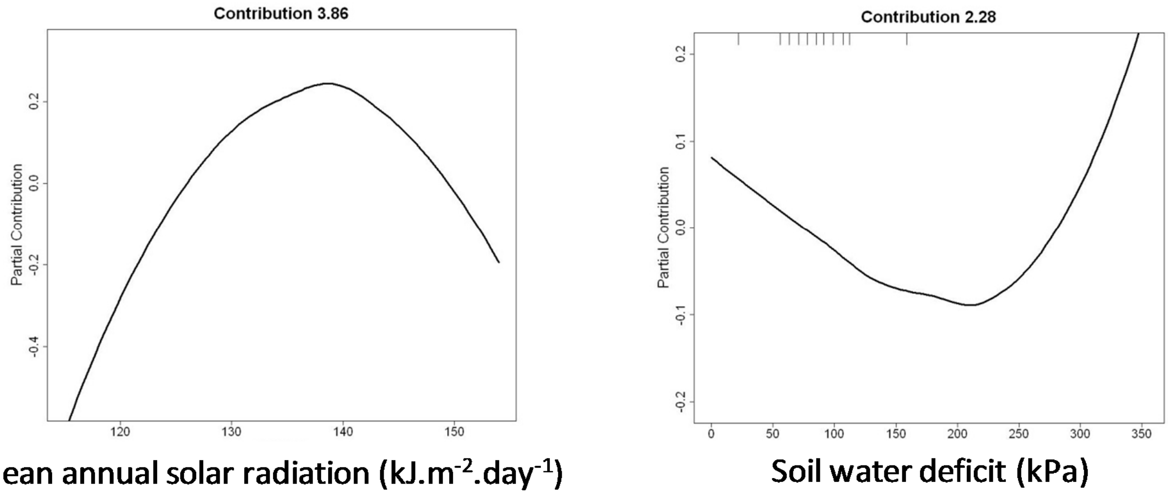

Fig S2b:

Figure S2 caption: Smoothed partial contributions of predictors retained in the final boosted regression tree model predicting tree species occurrence probability in non-forest survey plots. The contribution is the percentage of splits in the data made using each variable **–** regression trees are fitted by sequential dichotomous divisions of the data.
