# Supplementary figures and images for: Functional Traits Reveal Processes Driving Natural Afforestation at Large Spatial Scales
Source: PLoS One. 2013 Sep 18;8(9):e75219. doi: 10.1371/journal.pone.0075219 (PMC3776731; doi:10.1371/journal.pone.0075219)

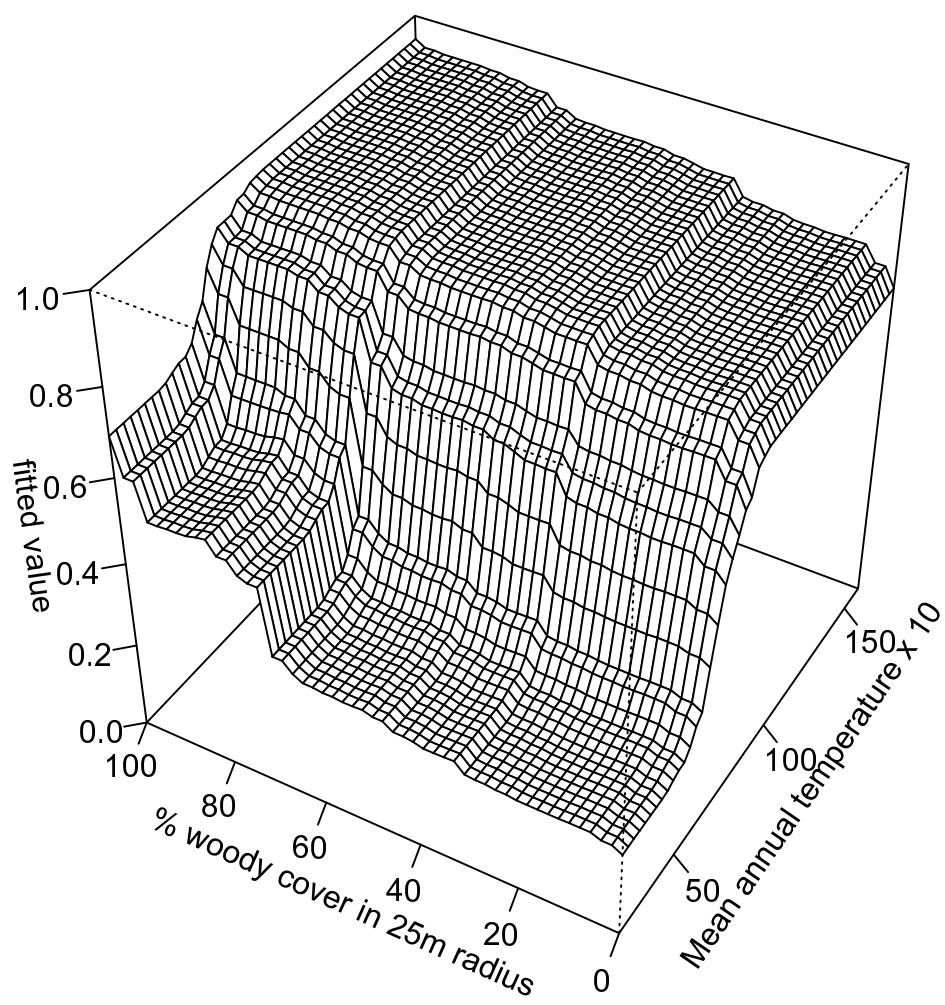

Supplement: Figure S3 — Perspective plot of predicted tree occurrence probability (fitted value) in final boosted regression tree model against local woody cover (% woody cover in 25 m radius) and mean annual temperature. (PDF) [file pone.0075219.s003.pdf]

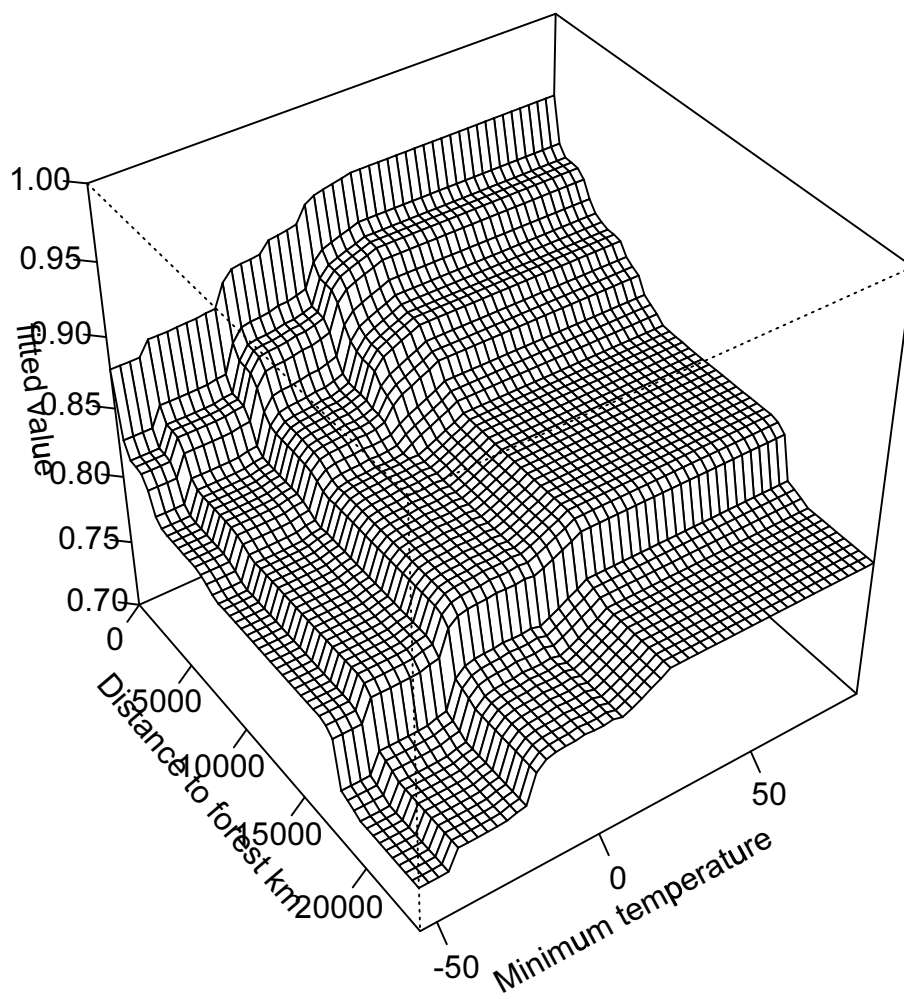

Supplement: Figure S4 — Perspective plot of predicted tree occurrence probability (fitted value) in final boosted regression tree model against distance to forest and minimum temperature. (PDF) [file pone.0075219.s004.pdf]
